# Long-term effects of straw return and straw-derived biochar amendment on bacterial communities in soil aggregates

**DOI:** 10.1038/s41598-020-64857-w

**Published:** 2020-05-12

**Authors:** Naling Bai, Hanlin Zhang, Sheng Zhou, Huifeng Sun, Yuhua Zhao, Xianqing Zheng, Shuangxi Li, Juanqin Zhang, Weiguang Lv

**Affiliations:** 1 Eco-environmental Protection Research Institute, Shanghai Academy of Agricultural Sciences, Shanghai, 201403 China; 2 Agricultural Environment and Farmland Conservation Experiment Station of Ministry Agriculture, Shanghai, 201403 China; 3 Institute of Biochemistry, College of Life Sciences, Zhejiang University, Hangzhou, 310058 China

**Keywords:** Ecology, Microbiology, Ecology, Environmental sciences

## Abstract

Improving soil structure, fertility, and production is of major concern for establishing sustainable agroecosystems. Further research is needed to evaluate whether different methods of straw returning determine the variations of soil aggregation and the microbial community in aggregates in the long term. In this study, we comparatively investigated the effects of long-term fertilization regimes performed over six years, namely, non-fertilization (CK), chemical fertilization (CF), continuous straw return (CS), and continuous straw-derived biochar amendment (CB), on soil aggregation and bacterial communities in rice-wheat rotation systems. The results showed that straw/biochar application increased soil nutrient content and soil aggregate size distribution and stability at both 0–20 cm and 20–40 cm soil depths, compared with those of CF and CK; CB performed better than CS. CB increased bacterial community diversity and richness in 0–20 cm soil, and evenness in 0–40 cm soil (*p* < 0.05); CS had no significant effect on these aspects. Variations in the relative abundance of Actinobacteria, Chloroflexi, Bacteroidetes, Nitrospirae, Gemmatimonadetes, and Latescibacteria in specific aggregates confirmed the different effects of straw/biochar on bacterial community structure. The partial least squares discrimination analysis and permutation multivariate analysis of variance revealed that fertilization, aggregate size fractions, and soil depth affected the bacterial community, although their effects differed. This study suggests that CB may reduce chemical fertilizer usage and improve the sustainability of rice-wheat cropping systems over the long term, with a better overall outcome than CS.

## Introduction

Aggregation is essential to provide the physical sheltering of organic matter and microbial inhabitants to maintain soil functions^1,2^. Soil organic carbon (SOC), aggregates, and soil microbiota are interrelated and interact with each other. SOC constitutes the key binding agent in the hierarchical architecture of aggregates, with its loss leading to aggregate degradation^3^. Microorganisms also carry out important functions in the formation and stabilization of soil aggregates, and their activities may differ in different aggregate fractions^4^. Garcia *et al*.^5^ noted that the basal microbial respiration in macroaggregates and the percentage of microaggregates within macroaggregates were indicative of SOC dynamics in soil. Thus, organic amendment generally alters soil aggregation, which may consequently affect the habit wherein microbes are heterogeneously distributed.

Rice-wheat rotation constitutes the primary cropping system in Southeast China, characterized by high chemical nitrogen (N) fertilizer input and high yields along with high straw production. Long-term application of inorganic fertilizers has been a driving force in soil structure deterioration, enzyme activity decrease, and concomitant soil fertility reduction^6,7^. Comprehensive utilization of agricultural residues could improve soil properties and facilitate soil aggregation process, ultimately restoring soil microenvironment^7,8^, and thereby sustaining crop productivity, which has attracted considerable attention during the last few decades.

Crop straw, which is rich in organic materials and soil nutrients with a low environmental footprint, can either be returned to the field directly or in the form of biochar^9^. Straw burning is currently forbidden, as it causes severe soil deterioration and air pollution^10^. Consequently, extensive utilization of crop straw has become a relevant concern, aside from mitigating the deleterious effects of long-term application of chemical fertilizers and promoting environmental sustainability. Straw return can usually maintain soil organic matter levels and increase soil aggregate stability^9^, thereby improving soil fertility and crop production^10^. Moreover, the content of SOC in each class of soil aggregate size can be significantly increased by straw return^11^. Nevertheless, other authors show that directly returning straw also has limitations, such as increasing greenhouse gas emissions and soil-borne diseases, affecting machinery tillage and seedling emergence, and causing unstable crop yields^12,13^. Sun *et al*.^14^ noted that wheat straw at two different application levels produced no detectable effects on bacterial community structure. Luo *et al*.^13^ also reported that straw fertilization decreased the diversity of the *Nitrospira*-like bacterial community (Shannon index) in the long-term. Biochar has the potential to serve as a soil conditioner, for example, by increasing soil aggregate size distribution and stability and C sequestration, improving pore-space status^15,16^, and reducing greenhouse gas emissions^17^. The effects of biochar incorporation may largely depend on the soil, biochar properties, and field management^15,18^. Jeffery *et al*.^19^ highlighted that irrespective of pyrolysis temperature and application rate, the soil water retention, aggregate stability, field saturated hydraulic conductivity were not affected by biochar amendment. Therefore, straw/biochar incorporation has shown no consistent effects on soil qualities and soil microbial communities.

Return of straw or biochar to the field is a common tactic to dispose of the mass of agro-waste in the rural areas of China, and some comparative studies have been reported. In a short-term experiment in the laboratory, the abundance of gram-positive bacteria and fungi was increased by straw treatment, whereas that of gram-negative bacteria was relatively high after the straw-derived biochar amendment^20^. Yang *et al*.^21^ reported that in a short-term field assay, straw-derived biochar application significantly decreased the accumulative emission of CO_2_ by 24%, compared with directly returning straw; straw-returning increased the carbon pool management index (*p* < 0.05); it omitted the shift in microbial communities, which were critically involved in nutrient cycling and soil structure. There are few long-term comparative studies on different approaches to return crop straw. The magnitude of organic matter-induced changes is dynamic; short-term changes may not be significantly indicative of longer-term conditions^22^. Therefore, a long-term experiment was designed to analyze and compare the effects of continuous straw/straw-derived biochar amendment on crop yield, soil physicochemical properties, soil aggregation, and the microbial community structure in aggregates.

## Results

### Grain yield and soil physicochemical properties

The grain yields obtained with fertilization (CF, CS, and CB treatments) significantly differed from those with CK (*p* < 0.05) in 2010–2016 (Table 1). The average annual yield in CF, CS, and CB treatments was 4.70, 4.54, and 4.66 t ha^−1^ (wheat) and 8.98, 9.13, and 9.09 t ha^−1^ (rice), respectively. The rice and wheat yield with fertilization treatments were approximately 2 and 2.5 times the yield with CK, respectively, with a trend of stability along with planting years. The physicochemical characteristics of soil samples are presented in Table 2. Generally, compared with CK, the incorporation of straw/biochar improved the soil nutrient content. The nitrate nitrogen (NO_3_^−^–N), ammonium nitrogen (NH_4_^+^–N), available phosphorus (AP), and SOC contents and the cation exchange capacity (CEC) differed significantly between the treatments in both 0–20 cm and 20–40 cm soil layers; the water content and pH exhibited significant differences only at 0–20 cm depth (*p* < 0.05). Soil available nutrients (AP, NO_3_^−^–N, and NH_4_^+^–N) were notably altered by fertilization in comparison with those in CK in both 0–20 cm and 20–40 cm soil layers. Fertilizer management tended to decrease soil pH in the 0–20 cm soil layer with CS showing a significant difference (*p* < 0.05).

**Table 1:** Rice/wheat yield under different fertilization treatments from 2010–2016 (t ha^−1^).

Grain	Treatments	2010	2011	2012	2013	2014	2015	2016
Wheat	CK	/	2.85(0.13)b	1.99(0.02)c	1.88(0.03)b	1.78(0.09)c	1.65(0.06)c	1.96(0.04)c
	CF	/	3.75(0.23)a	3.69(0.20)a	5.06(0.06)a	4.88(0.13)a	5.01(0.02)b	5.79(0.04)a
	CS	/	3.46(0.18)a	3.52(0.22)ab	5.00(0.16)a	4.44(0.12)b	5.25(0.17)a	5.59(0.09)b
	CB	/	3.55(0.13)a	3.35(0.05)b	5.16(0.03)a	4.69(0.32)ab	5.25(0.10)a	5.94(0.03)a
Rice	CK	5.26(0.13)b	4.06(0.20)b	4.28(0.16)c	4.39(0.10)b	4.62(0.13)c	3.75(0.06)c	/
	CF	8.85(0.13)a	8.90(0.05)a	9.45(0.11)a	9.97(0.07)a	9.59(0.17)b	7.14(0.07)b	/
	CS	8.72(0.25)a	8.87(0.06)a	9.25(0.08)b	10.20(0.25)a	9.97(0.13)a	7.79(0.05)a	/
	CB	8.58(0.10)a	8.86(0.11)a	9.34(0.05)ab	10.22(0.07)a	9.79(0.19)ab	7.75(0.23)a	/

Data represent the mean values with standard errors (n = 3). Different lowercase letters in the same season shown after the values indicate significant differences (ANOVA, *p* < 0.05). “/” indicates no data available.

**Table 2:** Physicochemical characteristics of soil samples under different fertilization treatments and soil depths.

Items	0–20 cm				20–40 cm			
	CK	CF	CS	CB	CK	CF	CS	CB
pH (H_2_O)	8.51(0.67)a	8.21(0.55)ab	8.01(0.42)b	8.60(0.24)ab	8.81(0.27)a	8.44(0.53)a	8.48(0.25)a	8.73(0.04)a
SOC (g kg^−1^)	7.98(1.51)b	9.09(0.70)b	9.61(3.27)ab	13.08(0.58)a	3.31(0.91)b	3.10(0.69)b	5.01(0.30)a	5.54(1.17)a
AP (mg kg^−1^)	30.49(0.72)b	31.91(3.58)ab	33.12(2.14)ab	35.35(2.83)a	29.85(0.39)bc	27.86(1.97)c	32.85(2.29)ab	34.71(3.42)a
NO_3_^−^–N (mg kg^−1^)	14.20(0.09)c	19.60(0.40)b	19.50(0.22)b	20.40(0.42)a	8.24(0.03)c	12.39(0.16)b	12.80(0.10)ab	13.87(0.13)a
NH_4_^+^–N (mg kg^−1^)	3.10(0.06)b	4.30(0.08)a	4.40(0.10)a	4.30(0.07)a	2.76(0.07)c	3.89(0.09)b	4.05(0.06)a	3.92(0.10)ab
Water content (%)	23.00(2.86)b	24.38(1.69)ab	27.28(2.44)a	26.75(1.76)ab	20.87(1.41)a	26.40(5.78)a	24.58(2.69)a	24.55(2.40)a
CEC (cmol kg^−1^)	14.61(0.89)c	20.61(1.15)b	25.38(0.75)a	26.92(0.35)a	11.16(0.27)c	16.54(1.15)b	22.18(0.78)a	23.45(0.87)a

Data represent the mean values with standard errors (n = 3). Different lowercase letters in the same soil depth and parameter shown after the values indicate significant differences (ANOVA, *p* < 0.05).

### Distribution and stability of soil water-stable aggregates

**Figure 1:**
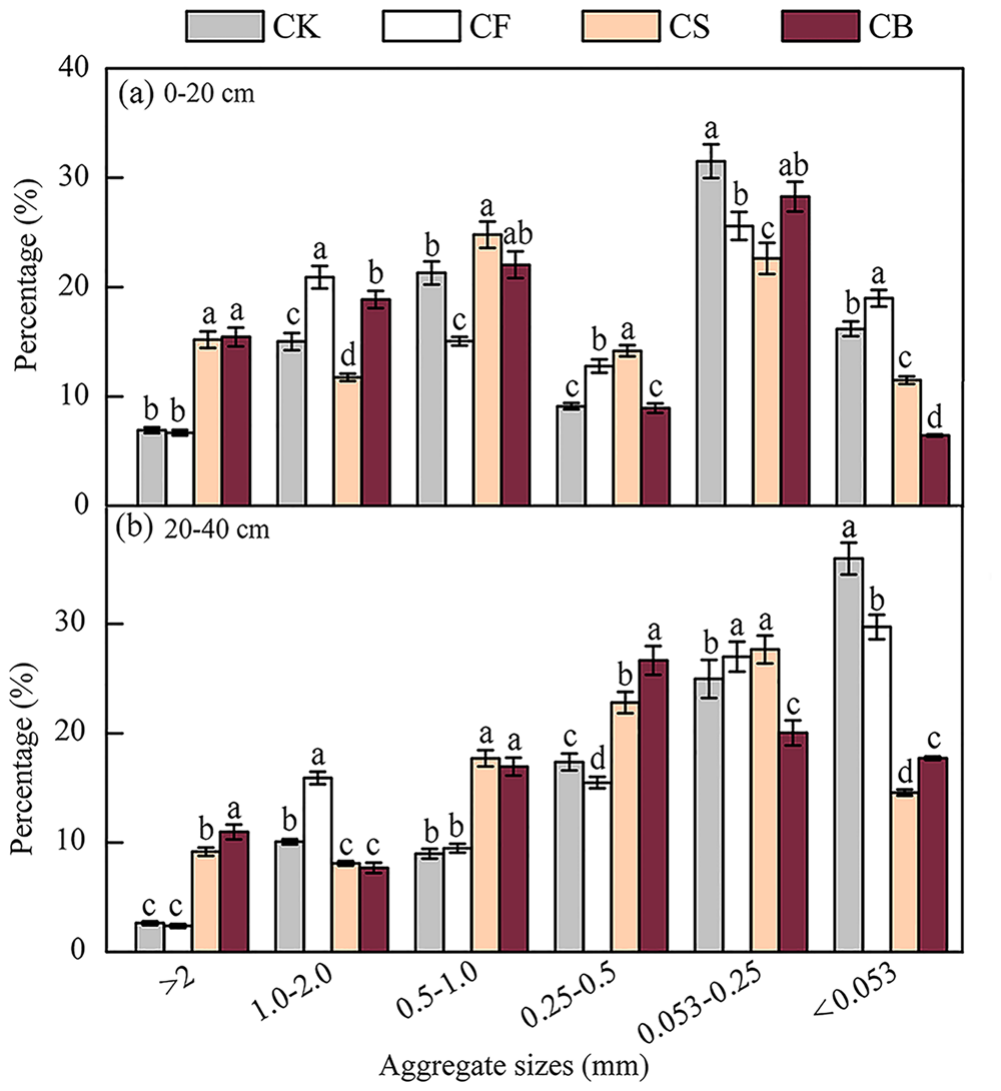
Effects of fertilization on soil water-stable aggregate distribution at 0–20 cm (**a**) and 20–40 cm (**b**) soil depths (using ORIGIN 9.0, OriginLab Corporation, Northampton, MA, USA). Different letters indicate significant differences at *p* < 0.05 between different treatments in specific aggregates.

**Figure 2:**
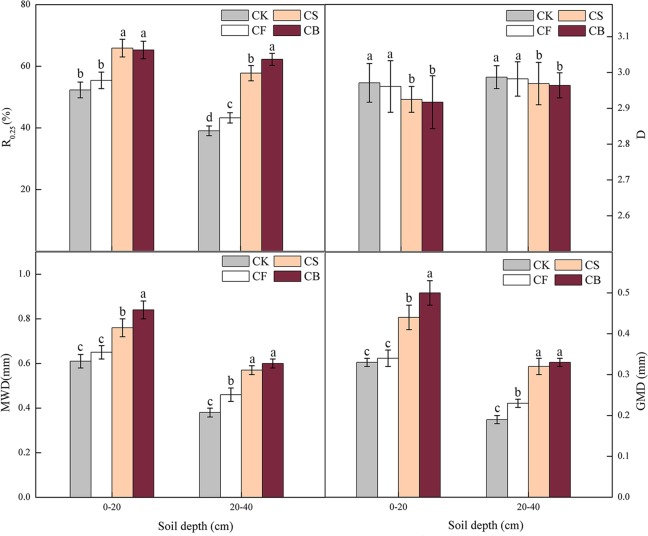
Effects of different treatments on the stability of water-stable soil aggregates (using ORIGIN 9.0, OriginLab Corporation, Northampton, MA, USA). R_0.25_: soil macroaggregates with a diameter larger than 0.25 mm, MWD: mean weight diameter, GMD: geometric mean diameter, D: fractal dimension. Different letters indicate differences at a 5% level of significance in the same soil depth.

Microaggregates (0.053–0.25 mm) and silt + clay (<0.053 mm) were dominant at 0–20 cm (27%) and 20–40 cm (25%) depths, respectively (Fig. 1). At 0–20 cm soil depth, CS improved the content of macroaggregates of size >2.0 mm (127%), 0.5–1.0 mm (65%), and 0.25–0.5 mm (11%), but decreased that of macroaggregates of size 1.0–2.0 mm (44%), microaggregates (12%), and silt + clay (40%), compared with those of CF (*p* < 0.05). In addition, CB increased the content of macroaggregates of size >2.0 mm (133%) and 0.5–1.0 mm (46%), and decreased the content of macroaggregates of size 1.0–2.0 mm (10%) and 0.25–0.5 mm(30%), and silt + clay (66%), as compared with those of CF (*p* < 0.05). A similar tendency was observed at 20–40 cm soil depth (Fig. 1b). Both CS and CB increased the content of macroaggregates of size >2.0 mm, 0.5–1.0 mm, and 0.25–0.5 mm, and decreased that of macroaggregates of size 1.0–2.0 mm and silt + clay, relative to those of CF and CK (*p* < 0.05). CB significantly decreased the ratio of microaggregates compared with those of CK, CF, and CS by 20%, 26%, and 28%, respectively (*p* < 0.05). Both CS and CB significantly increased the proportion of soil macroaggregates (R_0.25_) by 25–26% (top-layer soil) and 48–67% (deep-layer soil), respectively, compared with that of CF (*p* < 0.05) (Fig. 2). The effect of CB was more prominent than that of CS, suggesting that biochar could ameliorate soil structure to a more extensive degree.

With soil depth, the mean weight diameter (MWD) and geometric mean diameter (GMD) values significantly decreased; the fractal dimension (D) increased (Fig. 2). In the top-layer soil, the MWD under CB treatment was higher than that under the other three treatments by 11–38%; the MWD in CS was notably higher than that of CF and CK, but lower than that of CB (*p* < 0.05). At 20–40 cm depth, both CS and CB significantly increased the MWD value compared with CK and CF (*p* < 0.05). Similarly, the GMD value in CS and CB was significantly higher than that in the other two treatments (CK and CF) at 0–20 cm and 20–40 cm soil depths (*p* < 0.05). All the fertilization treatments produced higher MWD and GMD values at 0–20 cm compared with those at 20–40 cm soil. The variation in D was contrary to the trend of MWD and GMD; the D values under CS and CB were significantly lower than those under CF and CK at both 0–20 cm and 20–40 cm soil depths (*p* < 0.05).

### Bacterial alpha diversity in soil aggregates

The bacterial alpha diversity of aggregates under different fertilization regimes is shown in Table 3. With respect to macroaggregates at 0–20 cm soil depth, Shannon, Simpson, and Simpsoneven (a Simpson index-based measure of evenness) indices were increased in CB treatment, compared with those of other treatments. In microaggregates, CB also increased the Chao1, Simpson, and Simpsoneven indices, compared with those of CK (*p* < 0.05). In silt + clay, CS presented lower values of Chao1, Shannon, and Simpsoneven indices than CB treatment (*p* < 0.05). A similar tendency was also observed in the deeper soil layer (20–40 cm) for Simpson and Simpsoneven indices; the results indicated that CB treatment established a higher biodiversity and even bacterial community. In the present study, CB treatment yielded relatively high bacterial richness (Chao1) and diversity (Shannon and Simpson) at the 0–20 cm soil depth, and higher bacterial evenness (Simpsoneven) in both 0–20 cm and 20–40 cm layers, whereas CS had no significant effect on these aspects. The decreased bacterial alpha diversity at 20–40 cm soil depth was due to the reason that the availability of nutrients to microbes may be affected by soil depth^23^.

**Table 3:** Analysis of soil bacterial community alpha diversity at the 0–20 cm and 20–40 cm soil depths.

Soil depth (cm)	Alpha diversity indices	Macroaggregate				Microaggregate				Silt + clay			
		CK	CF	CS	CB	CK	CF	CS	CB	CK	CF	CS	CB
0–20	Chao1	4383a	4337a	4242a	4510a	4110b	4280ab	4275ab	4415a	4102b	4173ab	4143b	4476a
	Shannon	6.493b	6.636ab	6.422b	7.013a	6.630a	6.682a	6.618a	6.828a	6.561ab	6.359b	6.443b	6.948a
	Simpson	0.990ab	0.992ab	0.990b	0.996a	0.992b	0.994ab	0.993ab	0.996a	0.991a	0.983a	0.991a	0.995a
	Simpsoneven	0.039b	0.041b	0.035b	0.083a	0.042b	0.055ab	0.045b	0.078a	0.043b	0.030b	0.037b	0.092a
20–40	Chao1	3460a	4333a	3883a	4393a	3914a	4210a	3767a	4226a	3730a	3970a	3535a	4215a
	Shannon	6.106a	6.446a	6.066a	6.646a	6.360a	6.647a	5.884a	6.709a	6.186a	6.465a	5.964a	6.552a
	Simpson	0.984ab	0.989ab	0.982b	0.995a	0.986a	0.994a	0.936a	0.996a	0.983a	0.992a	0.973a	0.994a
	Simpsoneven	0.025b	0.034b	0.026b	0.069a	0.029b	0.053ab	0.064a	0.069a	0.035ab	0.060ab	0.022b	0.069a

The values indicate the average data for each index (n = 3). Different letters shown after the values indicate significant differences (ANOVA, *p* < 0.05).

### Bacterial beta diversity in soil aggregates

**Figure 3:**
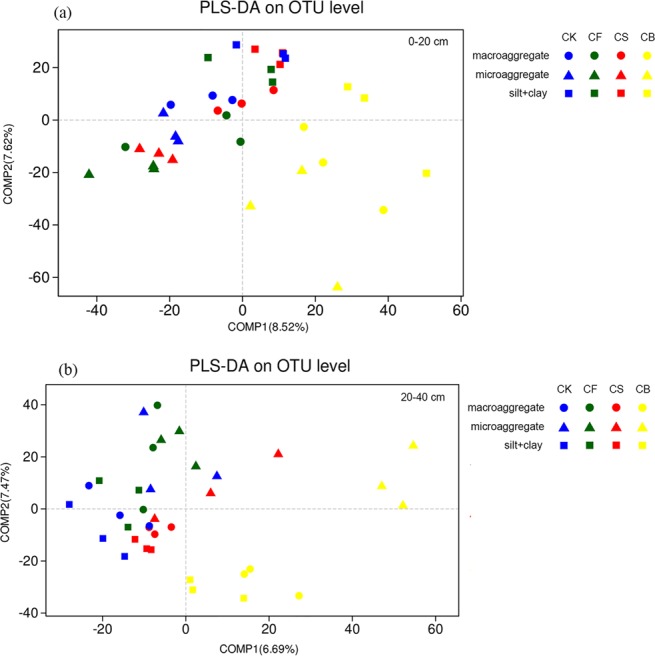
Partial least squares discrimination analysis (PLS-DA) showing the changes in bacterial community composition in different treatments in the 0–20 cm (**a**) and 20–40 cm (**b**) layers (using R V2.15.3). CK, CF, CS, and CB refer to different soil samples subjected to different treatments (i.e., non-fertilization, chemical fertilization, continuous straw returning, and continuous straw-derived biochar amendment, respectively).

The partial least squares discrimination analysis (PLS-DA) was performed to describe the similarity and dissimilarity of bacterial community structure with abundance standardization (Fig. 3). In the 0–20 cm soil layer, CB samples grouped at the right of the graph along the X axis, whereas other samples in CK, CF, and CS gathered at the top left and separated according to aggregate fractions (Fig. 3a). Aggregates with the same level were closer and more similar in CK, CF, and CS treatments, whereas the bacterial community structure of CB differed from that of the other treatments. COMP1 and COMP2 contributed 8.52% and 7.62%, respectively, to the changes in bacterial community composition at the 97% operational taxonomic unit (OTU) level. Similarly, at the 20–40 cm depth, samples under CB treatment and the other treatments tended to be distributed at the right and left part of the graph along COMP1 (6.69%), respectively (Fig. 3b). Furthermore, in 0–20 cm and 20–40 cm soil, soil aggregation exhibited more prominent roles in the bacterial distribution in CB treatment than in the other three treatments. Permutation multivariate analysis of variance (PERMANOVA) revealed that the bacterial community was significantly affected by fertilization treatments (18.2%) and soil aggregations (14.5%) at the 0–20 cm soil depth (Fig. S1a). In the deep-layer soil, the bacterial community was mainly altered by fertilization (19.3%) rather than soil fraction (8.6%) (Fig. S1b).

### Comparison of bacterial community composition

**Figure 4:**
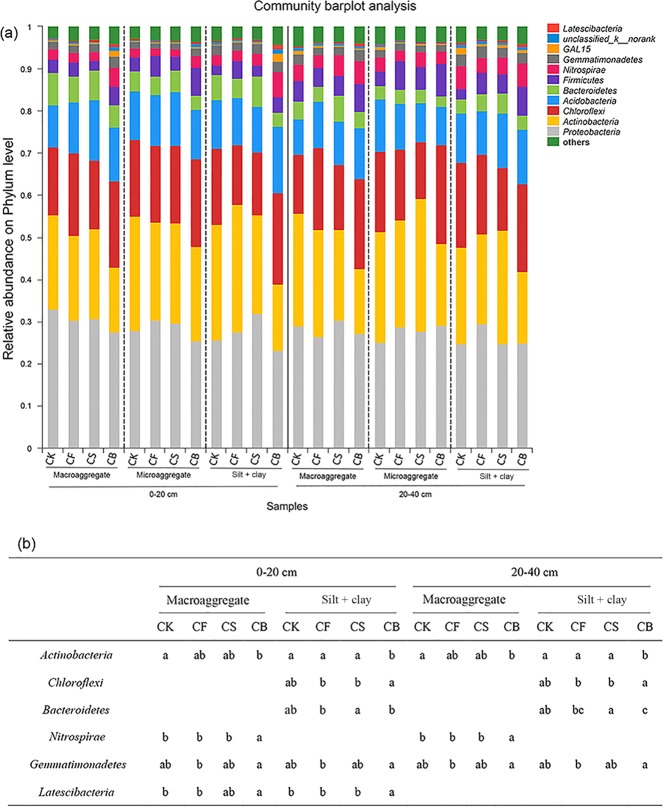
Relative abundances of the dominant bacteria at the phylum level (**a**) and the summarized phyla with statistical differences in each treatment in aggregates (**b**) (using R V2.15.3). Different letters at each aggregate fraction and each phylum indicate significant differences at *p* < 0.05 according to the least significant difference (LSD) test.

The dominant phyla were Proteobacteria, Actinobacteria, Chloroflexi, Acidobacteria, Bacteroidetes, Firmicutes, and Nitrospirae, accounting for more than 86% of the bacterial composition for each sample at both 0–20 cm and 20–40 cm soil depths (Fig. 4a). Proteobacteria and Actinobacteria were the two most abundant phyla identified in all the treatments. Fertilization regimes affected bacterial community composition at the phylum level to varying degrees (Fig. 4b). Under the present experimental conditions, the increase in the relative abundance of Chloroflexi, Nitrospirae, Gemmatimonadetes, and Latescibacteria and decrease in Actinobacteria and Bacteroidetes in specific aggregates were related to biochar amendment. No significant difference was observed between the treatments regarding these microbes in microaggregates at neither 0–20 cm nor 20–40 cm soil depths.

Fertilization management also affected bacterial community structure at the genus level, and the top 10 representative bacterial genera were listed in Tables S2 and S3. At 0–20 cm soil depth, compared to CF, the relative abundances of *Nitrospira* (macroaggregate) and *norank_f__Anaerolineaceae* (silt + clay) were increased; *Sphingomonas* (microaggregate), *Pseudarthrobacter* (silt + clay) were significantly decreased in CB. Meanwhile, *Pseudarthrobacter* (macroaggregate and microaggregate), *Nitrospira* (macroaggregate), *Sphingomonas* (microaggregate and silt + clay), and *norank_f__Anaerolineaceae* (silt + clay) showed significant difference between CS and CB treatments (*p* < 0.05). In the 20–40 cm soil layer, CB increased the relative abundances of *norank_f__Anaerolineaceae* (microaggregate) and *norank_c__Ardenticatenia* (microaggregate), compared with those in CF (*p* < 0.05). CS showed significant differences in the relative abundances of *Pseudarthrobacter* (silt + clay) and *norank_f__Anaerolineaceae* (microaggregate and silt + clay), as compared with those in CB (*p* < 0.05).

## Discussion

It is well understood that fertilization and soil quality significantly affect the productivity of grain crops. Compared with CK, the fertilization treatments (CF, CS, and CB) considerably increased crop yield (Table 1). Similar effects have been reported by Zhang *et al*.^9^, based on a 4-year field experiment with different straw application rates. Compared to CF, biochar treatment increased SOC content in both 0–20 cm and 20–40 cm soil significantly; directly returning straw increased the SOC content by 61.61% in 20–40 cm soil. Both biochar and straw application improved nutrient availability (AP, NO_3_^−^–N, and NH_4_^+^–N). The results indicated that the incorporation of organic compounds could improve soil structure and enhance the retention of nutrients, which was consistent with the findings of previous studies^24,25^. The decrease in soil pH at 0–20 cm soil depth upon CS treatment may be due to the indigenous organic acids released during straw decomposition^26^. Distance-based redundancy analysis (db-RDA) of bulk soil showed that CEC, SOC, AP, and NO_3_^−^–N were significant factors affecting bacterial distribution; CB treatment positively correlated with CEC changes (Fig. S2). In the present study, the soil health and resilience to retain nutrients were improved, with the maximum values of soil physicochemical properties being observed following CB treatment.

The MWD and GMD were used to reflect agglomeration aggregation and soil aggregate stability; the D value negatively correlated with soil permeability^27^. Generally, continuous conventional fertilization tends to disrupt aggregates, resulting in a decrease in the proportion of both macroaggregates and microaggregates^28^. Organic matter addition could increase the portion of macroaggregates and reduce microaggregates and silt + clay particles^29^; a similar phenomenon was also observed in the present study (Fig. 2). The increased soil aggregate stability in CS and CB treatments may correlate with the enhanced nutrient content in soil^1,30^ and the changes in microbial community structure^5^. Therefore, compared to conventional fertilizer management, agricultural waste return to the field was beneficial for improving soil aggregation and aggregate stability.

It is necessary to investigate further into the complicated relationships between habitat characteristics and the microbial community not only to understand soil biological functions for agricultural production but also to identify successful soil quality management. CS did no favor to bacterial diversity, richness, and evenness (Table 3). Similarly, Maarastawi *et al*.^31^ reported that microbial communities exhibited rather weak responses to rice straw application relative to other factors (i.e., crop rotation, oxygen availability, and field location). The underlying mechanisms are hypothesized to be: (i) straw returning to the field predominantly affects the fungi community as they release a broader range of extracellular enzymes to degrade different recalcitrant biopolymers in straw^20,32^; (ii) bacteria and fungi possibly dominated in the initial phase and later stage of crop residues decomposition, respectively^31^. In the present study, the biochar applied to soil was produced at low temperatures; thus, more soil organic matter and the labile components of the biochar could be co-mineralized by microorganisms^18^.

The interactive effect of fertilization and aggregation was rather significant (40.7%) at 0–20 cm soil depth on the bacterial community structure (Fig. S1), indicating that these factors together may have reshaped the bacterial community structure in the wheat harvest soils. In comparison, the bacterial community structure was mainly altered by fertilization (19.3%) in the deep-layer soil. Soil bacterial communities vary with fertilization, yet the detailed responses are frequently inconsistent and site-specific. For example, Liao *et al*.^33^ reported that both fertilization (54.6%) and soil fractions (21.2%) altered the bacterial community in 0–20 cm soil; however, the synergistic effect was deemed relatively weak (8.5%). The results in Fig. 3 indicated that both fertilization management and soil aggregation exerted certain effects on the bacterial community composition in the sandy loam soil of Eastern China. The variation of axes in Fig. 3 was relatively low, which might be related to the factors such as the application rate of inorganic and organic matter, soil inherent conditions, and the characteristics of the straw/biochar. The relatively high ratio of chemical fertilizer to straw/straw-derived biochar application in the field possibly decreased the significance of difference. More researches should be performed by reducing this proportion to explore the variation of soil bacterial communities in different aggregates.

Soil microbial communities play a vital role in maintaining soil structure and aggregation; microbial distribution differs with aggregate sizes since aggregates constitute a complex environment for microorganisms. The higher amount of available C in the biochar used in the present study, compared with that in commercial products, may explain the decrease in Actinobacteria in CB, whose abundance was supposed to be associated with the degradation of recalcitrant carbon compounds^8^. Bacteroidetes were suggested to be important in straw digestion as indicated in Fig. 4b; some Bacteroidetes species have been reported as important decomposers of hemicellulose or xylan^31^. Nitrospirae were primarily enriched in macroaggregates at both 0–20 cm and 20–40 cm soil depths in CB, indicating that soil nitrification in larger aggregates might be strengthened upon biochar incorporation^8^. Gemmatimonadetes, prevalent in drier soils^34^ such as the wheat planting soils, were enriched by CB treatment in macroaggregates and silt + clay (both at 0–20 cm and 20–40 cm). Latescibacteria present considerable capacity to degrade proteins, lipids, and polysaccharides^35^, thus facilitating N utilization and organic matter cycling. Notably, the Venn diagram analysis showed that the phylum Omnitrophica was specifically enriched in both CB (0–40 cm) and CS (20–40 cm) (data not shown). Omnitrophica, belonging to the Planctomycetes-Verrucomicrobia-Chlamydiae (PVC) superphylum, was probably involved in the removal of recalcitrant substances in the contaminated sites^36^.

Previous studies have already reported that organic amendment exhibited beneficial effects on microbial biomass, activity, and community structure of the soil^1,37^. The variation of the predominant top 10 genera in specific aggregate classes was noted in Tables S2 and S3; these preferences could strongly contribute to the spatial heterogeneity and bacterial diversity found in soils. The different responses of straw and biochar incorporation to soil were also observed by Pan *et al*.^20^, who found that straw shifted the microbial community structure to a distinct degree, whereas the results of the biochar and control treatments were overlapping. The inconsistency of the shifts in microbial communities in different aggregate classes can likely be attributed to the complex interactions between soil aggregation and microbial communities under different soil types and agricultural practices^38^. Moreover, the number of replicates was limited (n = 3) in the present field trial, which might partially affect the statistical analyses. Considering the complicated soil environment, more samples should be taken, if possible, to reduce the sampling error.

## Conclusion

The results of six years of fertilizer management experiments support our hypothesis that the application of straw and straw-derived biochar influences soil physicochemical characteristics, driving changes in crop production, and bacterial community structure. The nutrient availability, soil aggregate size distribution and stability in the 0–20 cm and 20–40 cm soil layers were enhanced by straw and biochar amendment, with the effect of biochar being more prominent. CB treatment increased soil bacterial diversity, richness, and evenness; CS had no significant effect on these aspects. Bacterial community composition and structure varied with soil depth, particle size fraction, and fertilization. The present study proposed that CB may reduce chemical fertilizer usage and improve soil sustainability, with better effects than CS. Nevertheless, it is important to consider other factors (e.g., the source of organic materials, application rate, plant growth periodicity, and other environmental factors) to achieve the maximum benefits and efficient use of straw and biochar in soil.

## Materials and methods

### Experimental design

The field experiment was conducted from June 2010 to May 2016 in the Low-Carbon Agricultural Engineering Technology Research Center, Zhuanghang Comprehensive Experimental Station, Shanghai, China (30°53′N, 121°23′E). The site is characterized by a subtropical climate with an average annual temperature of 15.8 °C and rainfall of 1178 mm. Rice (*Oryza sativa* L.) and wheat (*Triticum aestivum* L.) were rotated in the experimental field. The soil type was classified as sandy loam with an average SOC concentration of 8.50 g kg^−1^, total nitrogen (TN) concentration of 0.86 g kg^−1^, total phosphorus (AP) concentration of 0.52 g kg^−1^, available nitrogen (AN) concentration of 18.50 mg kg^−1^, AP concentration of 20.85 mg kg^−1^, CEC of 15.50 cmol kg^−1^, and pH of 8.33 in the 0–20 cm soil layer at the beginning of the experiment.

Four treatments were run in triplicate with a randomized complete block design, with each plot area being 60 m^2^. The crop varieties and management were the same apart from fertilization as follows: CK: non-fertilization; CF: chemical fertilizer application; CS: 3 t ha^−1^ straw directly returning; CB: 1 t ha^−1^ straw-derived biochar return. In the wheat growing season, 180, 90, and 204 kg ha^−1^ of pure N, P, and potassium (K) were applied, respectively; the application amount of pure N, P, and K was 225, 112.5, and 255 kg ha^−1^, respectively, during the whole rice-growing season. The same amount of total pure N, P, and K was applied in the CF, CS, and CB treatments; the deficiencies were implemented with inorganic fertilizers (Table S1). Inorganic N, P, and K fertilizers were urea, calcium superphosphate, and potassium sulfate, respectively. Pyrolysis was anaerobically performed at approximately 450–600 °C in a vertical charcoal furnace (ECO-5000, Zhejiang, China) to produce biochar. Soil was turned over for all treatments before each crop season planting, during which straw/straw-derived biochar was added into the soil. In detail, after rice/wheat harvest, straw/straw-derived biochar was spread on the soil surface as an amendment and thoroughly mixed with soil at approximately 15–20 cm depth prior to the subsequent wheat/rice crop planting. Therefore, all treatments had the same degree of soil disturbance.

### Soil sampling and measurement

Samples were collected from each plot after the wheat harvest on May 8, 2016. They were aseptically collected from 0–20 cm and 20–40 cm depths with a stainless-steel auger (15 mm interior diameter) using the five-point sampling method. The five pools in each plot were combined to provide one composite sample. Samples were placed into sterilized polyethylene sealed bags, stored at low temperature, and then immediately brought back to the laboratory. The fresh soil was gently peeled along natural planes of weakness; visible stones, roots, and other residues were removed. Approximately 100 g of each fresh soil sample was fractionated to different aggregate sizes (as described below) and subsequently frozen at −80 °C for bacterial community analysis.

Soil water-stable aggregates were briefly separated using an agglomerate analyzer following the wet sieving method^32,39,40^. The sieve apertures used here were 2.00, 1.00, 0.50, 0.25, and 0.053 mm. Soil samples in the sieve were slowly submerged in sterilized water for 5 min. The analyzer was then vertically shaken for 5 min at 50 times/min with the column kept in water. The fractions remaining on each sieve and aggregates of diameter <0.053 mm settled in the sieve barrel were respectively collected at the end of sieving. The parameters of R_0.25_, MWD, GMD, and D were calculated as follows^41,42^:

1 $${{\rm{R}}}_{0.25}=\frac{{{\rm{W}}}_{{\rm{r}} > 0.25}}{{{\rm{W}}}_{0}}$$

2 $${\rm{MWD}}=\mathop{\sum }\limits_{{\rm{i}}=1}^{{\rm{n}}}{{\rm{X}}}_{{\rm{i}}}{{\rm{W}}}_{{\rm{i}}}$$

3 $${\rm{GMD}}=\exp (\frac{{\sum }_{{\rm{i}}=1}^{{\rm{n}}}{{\rm{W}}}_{{\rm{i}}}{{\rm{lnX}}}_{{\rm{i}}}}{{\sum }_{{\rm{i}}=1}^{{\rm{n}}}{{\rm{W}}}_{{\rm{i}}}})$$

4 $$(3-{\rm{D}})\mathrm{lg}\left(\frac{{{\rm{X}}}_{{\rm{i}}}}{{{\rm{X}}}_{{\rm{\max }}}}\right)=\,{\rm{lg}}\,\frac{[{{\rm{W}}}_{({\rm{\delta }}\le {{\rm{X}}}_{{\rm{i}}})}]}{{{\rm{W}}}_{0}}$$

where, R_0.25_ is the proportion of aggregates with a diameter of >0.25 mm; X_i_ refers to the mean diameter of i-size aggregates; W_i_ is the dry weight of the i-size fraction collected relative to the total soil used; X_max_, W_(δ≤Xi)_, and W_0_ refer to the maximum diameter of all the aggregates tested, weight of aggregates with the particle-size <X_i_, and total weight of each size fraction, respectively. Therefore, D could be obtained by regression analysis.

Soil water content was measured gravimetrically and expressed as a percentage of soil water to dry soil with constant weight. Soil pH was measured (soil:water = 1:2.5) using a precision pH meter (METTLER TOLEDO, Shanghai, China). SOC was determined using an elemental analyzer (ELEMENTAR, Langenselbold, Germany) after complete removal of the inorganic carbon in soil by 1 M HCl^43^. NH_4_^+^–N and NO_3_^−^–N were assayed using Nessler’s reagent and the phenol disulfonic acid colorimetric methods, respectively. AP was measured using the ammonium molybdate ascorbic method after extraction with 0.5 M NaHCO_3_^7^. The CEC was determined using 1 M NH_4_OAc at pH 7.0 according to the protocol of the Analytical Methods of Soil Agricultural Chemistry^44^.

### Soil DNA extraction and high-throughput sequencing

The total genomic DNA was extracted from 0.5 g of soil aggregates using the MOBIO PowerSoil Soil DNA Isolation Kit and purified according to the manufacturer’s instructions. After quantification using a K5500 Micro-Spectrophotometer (KAIAO, Beijing, China), DNA was subjected to PCR amplification of the V3-V4 variable fragments of 16S rRNA with the primer set of 338 F (5′-ACTCCTACGGGAGGCAGCAG-3′) and 806 R (5′-GGACTACHVGGGTATCTAAT-3′)^45^. Sequencing was performed using Illumina MiSeq PE300 at Majorbio Bio-pharm Technology (Shanghai, China).

Quality filtering of the raw reads was performed to obtain high-quality clean reads according to Cutadapt (V1.9.1, http://cutadapt.readthedocs.io/en/stable/). Chimera sequences were identified and removed using the UCHIME algorithm (http://www.drive5.com/usearch/manual/uchime_algo.html), and clean reads were finally obtained. OTUs were clustered using UPARSE software (UPARSE v7.0.1001, http://drive5.com/uparse/) (97% similarity) and analyzed against the SILVA database. PCR product purification, library construction, and data processing and analysis were conducted as described previously^37^. Sequences (raw reads) have been deposited into the NCBI Sequence Read Archive (SRA) database (Accession Number: SRP148725).

### Statistical analysis

All comparative analyses were based on the normalized OTU abundance. Comparative analysis of soil physicochemical parameters and bacterial community structure between treatments was performed using a one-way ANOVA with the LSD test; results with *p* < 0.05 were considered statistically significant (SPSS 19.0, SPSS Corp., Chicago, IL, USA). The Chao1, Shannon, Simpson, and Simpsoneven indices were calculated using QIIME V1.9.1. PLS-DA, community barplot analysis, PERMANOVA (Bray-Curtis distances with 9999 permutations), and db-RDA (Weighted Unifrac) plots were generated using R V2.15.3. Other figures were produced using ORIGIN 9.0 (Originlab Corporation, Northampton, MA, USA).

### Statement

Field trials were designed to analyze the long-term effects of different fertilization approaches (blank control, conventional inorganic fertilization, straw returning, and straw-derived biochar amendment) on soil physicochemical properties, soil aggregation, crop production, and soil microenvironment, etc. Data about the soil aggregate size and physicochemical properties (Figs. 1, 2, and Table 2) were important for microbial community structure analysis. This manuscript quoted some relevant data of PeerJ (doi:10.7717/peerj.6171).

## Supplementary information

Supplementary information.
