# Supplementary material for: Long-term effects of straw return and straw-derived biochar amendment on bacterial communities in soil aggregates
Source: Sci Rep. 2020 May 12;10:7891. doi: 10.1038/s41598-020-64857-w (PMC7217948; doi:10.1038/s41598-020-64857-w)
Supplement: Supplementary file 1 — Supplementary information. [file 41598_2020_64857_MOESM1_ESM.pdf]

Long-term effects of straw return and straw-derived biochar amendment on bacterial communities in soil aggregates

Naling Bai[1,2†], Hanlin Zhang[1,2†], Sheng Zhou[1,2], Huifeng Sun[1,2], Yuhua Zhao[3], Xianqing Zheng[1], Shuangxi Li[2], Juanqin Zhang[2], Weiguang Lv[1,2*]

[1] Eco-environmental Protection Research Institute, Shanghai Academy of Agricultural Sciences, Shanghai 201403, China

[2] Agricultural Environment and Farmland Conservation Experiment Station of Ministry Agriculture, Shanghai 201403, China

[3] Institute of Biochemistry, College of Life Sciences, Zhejiang University, Hangzhou 310058, China

[†] The first two authors contributed equally to this work.

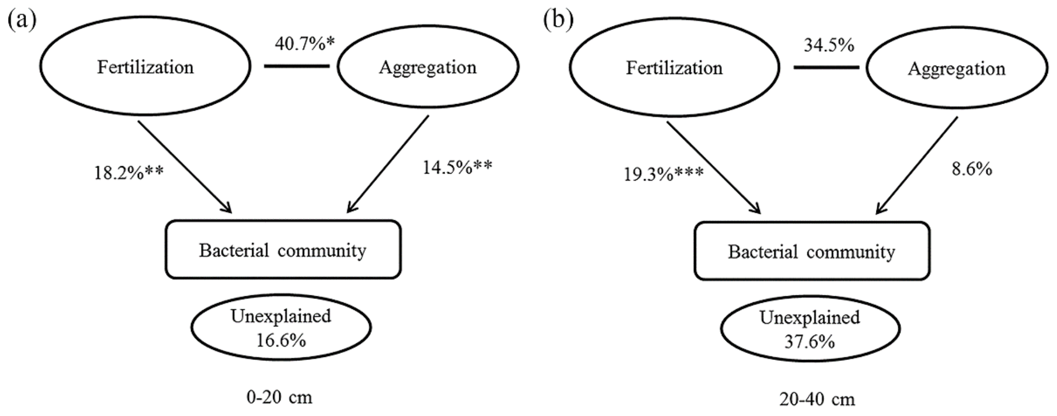
(a)
Fertilization
40.7%*
Aggregation
18.2%**
14.5%**
Bacterial community
Unexplained
16.6%
0-20 cm
(b)
Fertilization
34.5%
Aggregation
19.3%***
8.6%
Bacterial community
Unexplained
37.6%
20-40 cm

Figure S1

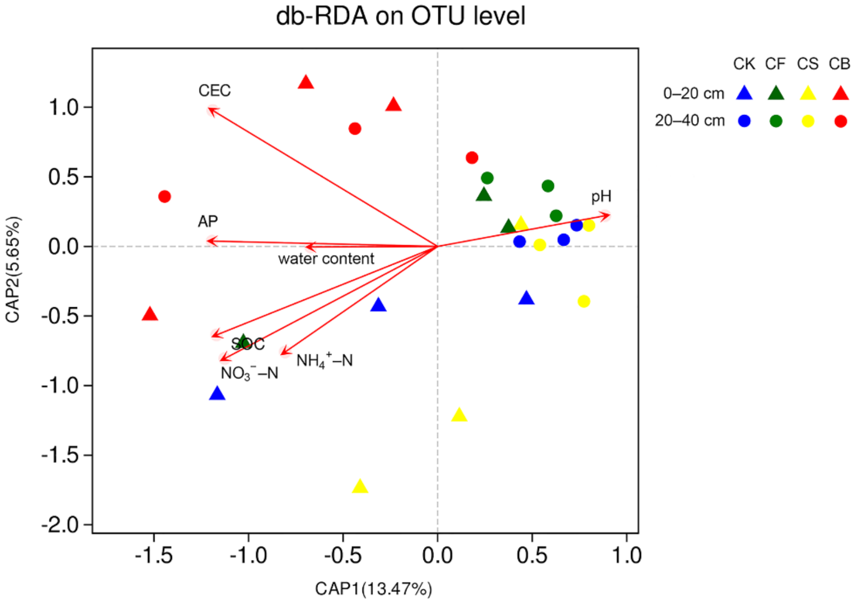
db-RDA on OTU level
CK CF CS CB
0–20 cm
20–40 cm
CEC
AP
water content
pH
SOC
NO3−–N
NH4+–N
CAP1(13.47%)
CAP2(5.65%)
1.0
0.5
0.0
-0.5
-1.0
-1.5
-2.0
-1.5
-1.0
-0.5
0.0
0.5
1.0

Figure S2

Table S1 Properties of straw/straw-derived biochar and inorganic fertilizers supplemented in different treatments.

Planting season	Organics application	Application rate ($t\ ha^{-1}$)	Nutrient content (%)			Inorganic fertilizers supplement ($kg\ ha^{-1}$)		
			N	P	K	N	P	K
Rice	Wheat straw	3.0	0.38	0.24	1.84	213.6	105.3	169.8
	wheat straw-derived biochar	1.0	1.71	0.64	4.72	207.9	106.1	177.8
Wheat	Rice straw	3.0	0.33	0.19	1.35	170.1	84.3	163.5
	rice straw-derived biochar	1.0	0.84	0.52	5.30	171.6	84.8	151.0

The characteristics of straw/straw-derived biochar list represent the average values for six years. N: nitrogen, P: phosphorus, K: potassium.

Table S2. Relative abundance of the top 10 dominant bacterial genera at the 0–20 cm soil depth.

Items	Macroaggrergate				Microaggregate				Silt + clay			
	CK	CF	CS	CB	CK	CF	CS	CB	CK	CF	CS	CB
Pseudarthrobacter	6.01ab	5.70ab	6.63a	2.99b	7.78a	5.66ab	6.58a	3.67b	7.62ab	10.97a	6.71ab	3.48b
norank_c__Acidobacteria	5.62a	7.71a	9.57a	7.82a	7.46a	8.09a	7.87a	7.77a	7.03a	7.19a	5.97a	11.15a
norank_f__Anaerolineaceae	4.97b	7.95a	5.68ab	6.92ab	6.49a	7.14a	8.03a	7.96a	5.32ab	3.88b	4.70b	8.48a
H16	1.18a	1.15a	0.99a	1.59a	1.16a	1.16a	0.94a	1.41a	1.20a	1.54a	1.43a	1.46a
Sphingomonas	3.93a	3.63a	4.33a	2.58a	2.81ab	3.20a	3.27a	1.32b	3.76ab	4.53ab	6.48a	2.19b
Nitrospira	2.44b	2.13b	2.15b	4.54a	2.11a	1.78a	1.69a	2.79a	2.50a	2.45a	2.71a	5.51a
unclassified_f__Intrasporangiaceae	2.24a	2.01a	2.39a	2.32a	3.18a	2.09bc	2.72ab	1.56c	2.76a	3.08a	2.45a	1.76a
Roseiflexus	2.77a	3.03a	2.59a	2.32a	3.71a	3.22a	3.20a	3.16a	3.19a	2.05a	2.02a	2.15a
Nocardioides	2.21a	1.59ab	1.63ab	0.99b	1.79a	2.31a	1.98a	3.01a	3.05a	2.81a	2.18a	1.38a
norank_f__Gemmatimonadaceae	1.34a	1.21a	1.39a	1.99a	1.16a	1.04a	1.08a	1.37a	1.62a	1.28a	1.73a	1.77a

CK, CF, CS, and CB refer to different soil samples subjected to different treatments. Different lowercase letters at each aggregate size and each genus indicate significant differences (ANOVA, $p < 0.05$).

Table S3. Relative abundance of the top 10 dominant bacterial genera at the 20–40 cm soil depth.

Items	Macroaggregate				Microaggregate				Silt + clay			
	CK	CF	CS	CB	CK	CF	CS	CB	CK	CF	CS	CB
Pseudarthrobacter	11.01a	8.35ab	10.09ab	3.94b	10.71a	6.25a	18.10a	4.00a	10.05ab	4.20ab	13.95a	3.70b
norank_c__Acidobacteria	4.81a	7.38a	6.94a	7.11a	8.71a	7.32a	6.43a	6.03a	7.13a	6.57a	9.33a	8.37a
Nitrospira	4.16a	2.74a	4.82a	5.32a	3.18a	2.45a	3.41a	2.95a	5.40a	3.57a	4.18a	5.57a
norank_f__Anaerolineaceae	2.85b	6.57ab	4.40ab	8.28a	7.07b	5.30b	4.51b	11.64a	6.28ab	4.89ab	3.92b	7.36a
H16	3.13a	1.24b	2.07ab	2.35ab	1.66a	1.45a	2.13a	1.85a	2.46a	1.47a	2.11a	2.54a
Roseiflexus	2.52a	3.57a	2.89a	2.50a	2.80a	3.66a	2.42a	3.39a	2.38a	4.20a	2.70a	2.60a
norank_c__Ardenticatenia	1.37a	1.26a	1.52a	1.97a	2.09ab	1.46b	1.85ab	3.13a	1.39a	1.30a	1.15a	1.74a
norank_c__Actinobacteria	1.69a	1.62a	1.19a	1.25a	2.03a	1.25ab	0.91b	1.12ab	2.08a	1.32a	1.58a	2.05a
Gaiella	1.45a	1.26a	1.06a	1.26a	1.26ab	1.94a	1.12b	1.65ab	1.12a	1.80a	1.15a	1.46a
norank_f__Gemmatimonadaceae	2.02a	1.34a	1.48a	2.07a	1.37a	1.25a	1.23a	1.08a	2.21a	1.98a	1.63a	1.81a

CK, CF, CS, and CB refer to different soil samples subjected to different treatments. Different lowercase letters at each aggregate size and each genus indicate significant differences (ANOVA, $p < 0.05$).
